# Occult Pathology in the Contralateral Prophylactic Mastectomy Specimen Despite a Negative Contralateral MRI: A Single-Center Cohort Study

**DOI:** 10.3390/cancers18162710

**Published:** 2026-08-21

**Authors:** Osman Cem Yılmaz, Adnan Gündoğdu, Merve Aktaş, Kübra Ertekin, Merve Tokoçin, Damiano Gentile, Ceyda Sönmez Wetherilt, Levent Çelik

**Affiliations:** 1 Breast Surgery, Private Clinic of Dr. Osman Cem Yılmaz, İstanbul 34740, Türkiye; 2 Department of General Surgery, Sancaktepe Şehit Prof. Dr. İlhan Varank Training and Research Hospital, İstanbul 34785, Türkiye; 3 Department of Surgical Oncology, Sakarya University Training and Research Hospital, Sakarya 54100, Türkiye; 4 Department of General Surgery, Faculty of Medicine, Afyonkarahisar Health Sciences University, Afyonkarahisar 03030, Türkiye; 5 Department of General Surgery, Bağcılar Training and Research Hospital, İstanbul 34200, Türkiye; 6 Breast Unit, IRCCS Humanitas Research Hospital, Rozzano, 20089 Milan, Italy; 7 Department of Medical Pathology, Cerrahpaşa Faculty of Medicine, İstanbul University-Cerrahpaşa, İstanbul 34098, Türkiye; 8 Department of Radiology, Radiologica Imaging and Diagnostic Center, İstanbul 34846, Türkiye

**Keywords:** contralateral prophylactic mastectomy, occult malignancy, high-risk lesion, breast MRI, BI-RADS, B3 lesion

## Abstract

Background/Objectives: Contralateral prophylactic mastectomy (CPM) is increasingly performed despite negative preoperative imaging. We evaluated the prevalence and MRI detectability of occult pathology in the contralateral breast. Methods: In this single-center retrospective cohort, 82 patients with unilateral invasive breast cancer underwent simultaneous CPM after preoperative contralateral MRI. Occult findings were classified as occult malignancy, atypical/high-risk lesions, or other lesions of uncertain malignant potential (B3 lesions); the primary outcome was clinically significant occult pathology (malignancy or an atypical/high-risk lesion). Proportions are reported with exact 95% confidence intervals and associations with exact odds ratios and Benjamini–Hochberg correction. Results: Occult malignancy occurred in 1/82 (1.2%; a single DCIS), clinically significant occult pathology in 14/82 (17.1%) and any occult pathology in 21/82 (25.6%). Among 59 patients with a negative MRI (BI-RADS 1–2), clinically significant occult pathology occurred in 18.6% and atypical/high-risk lesions in 16.9% (whole cohort, 15.9%). The single occult malignancy arose in an MRI-negative breast. No factor remained significant after correction for multiple comparisons. Conclusions: After preoperative MRI, occult malignancy is rare, whereas atypical and high-risk lesions frequently remain occult; a negative contralateral MRI excluded neither. These findings support individualized, shared decision-making rather than the yield of occult pathology as the basis for CPM.

## Simple Summary

Many women treated for cancer in one breast choose to have the other, healthy breast removed at the same time to reduce worry about a future cancer, and they usually have an MRI scan of that breast beforehand. We studied 82 such women to find out how often hidden disease was present in the removed healthy breast, and whether a normal MRI ruled it out. Hidden cancer was very rare (one of 82 women), and, when it did occur, it was in a breast with a normal MRI. Early warning changes that are not cancer but raise future risk were far more common and were usually invisible on MRI. These findings suggest that the decision to remove a healthy breast should rest on a woman’s personal risk and preferences rather than on the chance of finding hidden disease.

## 1. Introduction

The use of contralateral prophylactic (risk-reducing) mastectomy (CPM) in women with unilateral breast cancer has increased in recent years, and its appropriateness in average-risk patients continues to be debated [1,2]. For most patients CPM confers no survival benefit, and the annual risk of contralateral breast cancer for average-risk women generally remains low at approximately 0.1% to 0.6% per year, falling to 0.2–0.5% per year in those receiving adjuvant systemic therapy [2]. Current consensus recommendations discourage CPM in average-risk women and reserve it for defined high-risk situations, such as carriers of pathogenic BRCA1/2 variants, women whose family history confers a lifetime breast cancer risk above 25% in the absence of a pathogenic variant, or those with a history of mantle-field chest irradiation before age 30 [2]. In practice, however, the decision is often shaped by cancer-related anxiety, genetic test results, and imaging findings, raising the question of whether CPM represents a form of overtreatment in average-risk patients [1].

Professional societies converge on this position, with differences in emphasis. In North America, the American Society of Breast Surgeons states that CPM should be discouraged in average-risk women with unilateral breast cancer and reserves it for defined high-risk situations, and the Society of Surgical Oncology likewise confines the indication to women at high risk of contralateral breast cancer through a germline pathogenic variant, prior chest irradiation or a strong family history [2,3]. The European guidance is comparable: the ESMO Clinical Practice Guideline identifies breast-conserving surgery with postoperative radiotherapy as the preferred local treatment for the majority of patients with early breast cancer [4], and the 2025 St Gallen consensus panel recommended strong consideration of contralateral risk-reducing mastectomy for carriers of pathogenic BRCA1/2 variants, consideration of such surgery in PALB2 carriers, and intensified surveillance for lower-penetrance variants in ATM, CHEK2, BARD1 or RAD51 [5]. Data from Asia point in the same direction while illustrating how practice varies: in Korea the number of contralateral risk-reducing mastectomies in affected BRCA1/2 carriers rose approximately 5.8-fold between 2013 and 2017, with rates ranging from 2.9% to 44.4% across institutions [6], and a Japanese Hereditary Breast and Ovarian Cancer registry analysis found occult cancer in six of 53 prophylactic mastectomy specimens (11.3%) despite preoperative mammography, ultrasound and MRI [7]. The Turkish series have likewise examined the yield of occult disease at CPM and its implications for axillary staging [8].

In this context, preoperative breast magnetic resonance imaging (MRI) is increasingly used. In women with newly diagnosed breast cancer, MRI can detect clinically and mammographically occult contralateral malignancies [9,10] and, in the specific setting of the contralateral breast, offers a high negative predictive value for malignancy [11,12]. A negative contralateral MRI is therefore considered reassuring, although MRI findings themselves may also influence the decision to undergo CPM [9]. Nevertheless, many women elect CPM despite negative contralateral imaging because of cancer-related anxiety, family history, genetic susceptibility, or a desire for symmetry and peace of mind [1].

Even so, occult pathology, comprising both occult malignancy and high-risk lesions or lesions of uncertain malignant potential (B3 lesions), continues to be found in CPM specimens despite negative preoperative imaging [13,14]. A recent meta-analysis reported a pooled incidence of occult invasive malignancy of 1.55% in CPM specimens [14], and in high-penetrance variant carriers, occult malignancy is found in approximately 4% of prophylactic mastectomies, falling to about 3% when the preoperative MRI is reported as BI-RADS 1–2 [13]. Importantly, a substantial proportion of lobular carcinoma in situ, atypical lobular hyperplasia, atypical ductal hyperplasia, and other B3 lesions do not exhibit characteristic enhancement and may therefore remain occult on MRI [15]. A negative MRI thus does not exclude clinically relevant precursor lesions; yet data on the occult pathology that remains in the MRI-negative contralateral breast, and on the factors that predict it, are limited.

In this study, we aimed to determine, in patients with a preoperative contralateral MRI who underwent CPM, (i) the prevalence of occult pathology, (ii) the diagnostic performance of contralateral MRI for predicting occult pathology, and (iii) the factors associated with occult pathology, particularly in the MRI-negative breast. By clarifying what a negative contralateral MRI does and does not exclude, we sought to inform the ongoing discussion of the benefit-versus-overtreatment balance in this patient group.

## 2. Materials and Methods

### 2.1. Study Design

This single-centre, retrospective cohort study included patients who underwent surgery performed by a single breast surgeon at a clinic dedicated to breast surgery between January 2022 and December 2025. The study was approved by the institutional ethics committee (approval no. 2026/21, dated 14 January 2026) and was conducted in accordance with the principles of the Declaration of Helsinki. Owing to the retrospective design, the requirement for informed consent was waived. The study was reported in accordance with the Strengthening the Reporting of Observational Studies in Epidemiology (STROBE) guidelines [16]; the completed checklist is provided in Table S1.

### 2.2. Patient Selection

**Figure 1:**
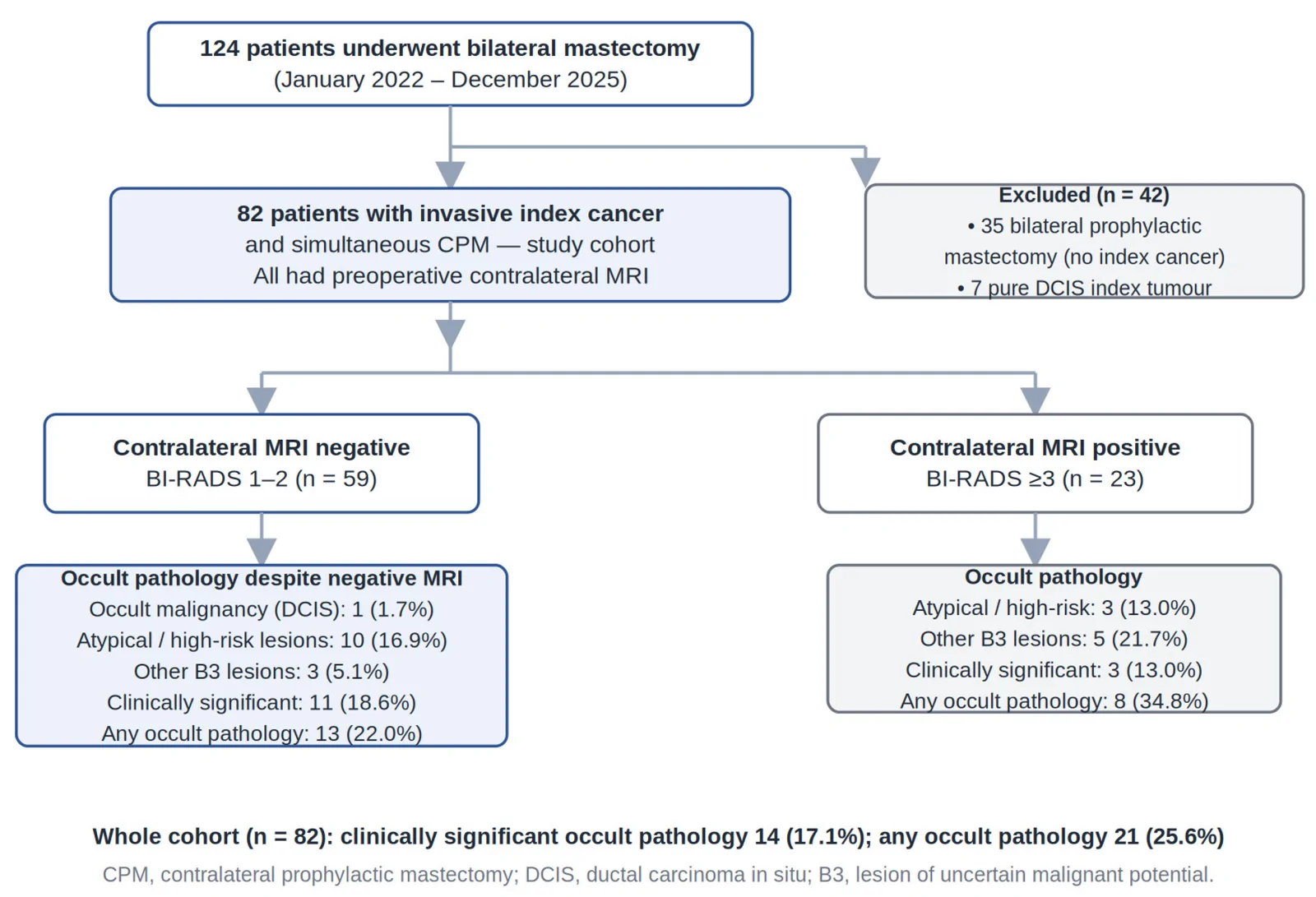
Patient selection and study flow. Of 124 patients undergoing bilateral mastectomy, 42 were excluded (35 bilateral prophylactic mastectomies without an index cancer and 7 with a pure DCIS index tumour), leaving 82 patients with invasive index cancer and simultaneous contralateral prophylactic mastectomy (CPM). All 82 had a preoperative contralateral MRI; 59 were MRI-negative (BI-RADS 1–2) and 23 MRI-positive (BI-RADS ≥ 3). Occult pathology findings are shown for each subgroup and for the whole cohort.

A total of 124 patients who underwent bilateral mastectomy at our clinic during the study period were identified. Of these, 35 underwent bilateral prophylactic mastectomy without an index cancer, and 7 had a non-invasive (pure ductal carcinoma in situ) index tumour; these 42 patients were excluded. The remaining 82 patients, who underwent therapeutic mastectomy of the index breast for unilateral invasive breast cancer with simultaneous contralateral prophylactic mastectomy (CPM) in the same operative session, constituted the study cohort. All 82 patients had undergone preoperative contralateral breast MRI. Patients whose contralateral breast was assessed as negative on MRI (BI-RADS 1–2, *n* = 59) were analyzed separately as a predefined subgroup to evaluate occult pathology detected despite negative imaging. The diagnostic performance of contralateral MRI was assessed in the entire cohort (*n* = 82). The patient selection flow is summarized in Figure 1.

### 2.3. Surgery and Pathological Evaluation

All patients underwent simultaneous contralateral prophylactic mastectomy. Surgical specimens were fixed in 10% neutral buffered formalin and serially sectioned at 5–10 mm intervals. Representative samples were obtained from each quadrant, from the nipple–areolar region and from any macroscopically suspicious area; in cases without a gross lesion, systematic sampling of the fibroglandular tissue was performed. Our sampling protocol did not mandate a fixed minimum number of blocks per specimen; the extent of sampling lay within the range described in published prophylactic mastectomy series, in which representative sections from each quadrant and the nipple are typical and for which no standard protocol exists [7]. All slides were evaluated as part of routine diagnostic practice by three different pathologists experienced in breast pathology, two with more than 10 years and one with more than 5 years of experience. Specimens were not re-reviewed centrally for the present study, and inter-observer agreement was not formally assessed. Lesions were classified according to the World Health Organization (WHO) Classification of Breast Tumours [17].

Contralateral occult findings were classified into three predefined tiers. Occult malignancy comprised occult invasive carcinoma and/or ductal carcinoma in situ (DCIS). Atypical/high-risk lesions comprised atypical ductal hyperplasia (ADH), atypical lobular hyperplasia (ALH), lobular carcinoma in situ (LCIS), flat epithelial atypia (FEA), and atypical papilloma. Other B3 lesions comprised radial scar/complex sclerosing lesion, papilloma without atypia, and phyllodes tumour; the atypical/high-risk and other B3 tiers together correspond to the B3 category defined by the Third International Consensus Conference on B3 lesions [15]. High-risk lesions such as atypical hyperplasia and lobular carcinoma in situ confer an increased risk of subsequent breast cancer [18,19,20]. The primary outcome, clinically significant occult pathology, was defined as occult malignancy or an atypical/high-risk lesion (Tier 1 + Tier 2); any occult pathology (Tiers 1–3) was a secondary outcome. Benign incidental findings (fibroadenoma, apocrine metaplasia, sclerosing adenosis, cysts, and other benign changes) were recorded but not counted as occult pathology.

### 2.4. Breast Magnetic Resonance Imaging

Breast MRI examinations were performed with the patient in the prone position using a 32-channel dedicated breast coil (Siemens Healthineers, Erlangen, Germany). Seventy-six of the 82 examinations were performed on a 1.5 T system (MAGNETOM Altea, Siemens Healthineers, Erlangen, Germany) at a single dedicated breast imaging centre; the remaining six were obtained at external centres before referral, and the equipment used at those centres is not known. The protocol comprised fat-suppressed T2-weighted images, unenhanced T1-weighted images, and both dynamic ultrafast and conventional dynamic contrast-enhanced fat-suppressed T1-weighted sequences. A gadolinium-based contrast agent (gadobutrol) was administered intravenously at a dose of 0.1 mmol/kg, and multiphase dynamic series with subtraction images were generated. All examinations, including those obtained at external centres, were interpreted prospectively, as part of routine clinical care, by the same two radiologists, each with more than 10 years of experience in breast imaging, and were reported according to the ACR BI-RADS MRI lexicon. In line with standard practice, readers had access to the clinical history, the index tumour findings and previous conventional imaging; the reports were not blinded, re-read or centrally reviewed for the purposes of this study, and the BI-RADS categories analyzed here are those recorded in the original prospective reports. The contralateral breast was assigned a BI-RADS category (1–5). BI-RADS 1–2 was considered a negative MRI result and BI-RADS ≥ 3 (BI-RADS 3, 4, and 5) a positive MRI result. BI-RADS 3 lesions were included in the positive group because they require short-interval follow-up or further assessment and may influence surgical decision-making; a sensitivity analysis classifying BI-RADS 3 as negative yielded consistent results.

### 2.5. Statistical Analysis

Descriptive statistics are presented as median (interquartile range, IQR) for continuous variables and count and percentage for categorical variables. Proportions were calculated with the Clopper–Pearson exact 95% confidence intervals (CI). Categorical comparisons used Fisher’s exact test and continuous variables the Mann–Whitney U test. Predictive associations were assessed with the exact conditional odds ratio (OR) and 95% CI; the Benjamini–Hochberg false discovery rate (FDR) correction was applied for multiple comparisons. An MRI-negative breast was defined as a contralateral MRI of BI-RADS 1–2; a BI-RADS 1–3 definition was additionally evaluated as a sensitivity analysis. Given the low event counts, all predictor analyses were considered exploratory and hypothesis-generating, and no multivariable modelling was performed. A two-sided *p* < 0.05 was considered statistically significant. Analyses were performed in Python 3.12 (SciPy, statsmodels).

## 3. Results

### 3.1. Cohort Characteristics

Eighty-two patients with unilateral invasive breast cancer who underwent simultaneous CPM were included. The median age was 42 years (IQR 38–48; range 26–80), and 65/82 (79%) were premenopausal. The index tumour was invasive ductal carcinoma in 70, invasive lobular in nine, and mixed type in three patients. A BRCA1/2 pathogenic variant was present in 19 of 60 tested patients (32%; BRCA1, 10; BRCA2, 9). All 82 patients had a preoperative contralateral breast MRI, which was negative (BI-RADS 1–2) in 59 (72%). Cohort characteristics and their association with clinically significant occult pathology are shown in Table 1.

### 3.2. Contralateral Occult Pathology

**Figure 2:**
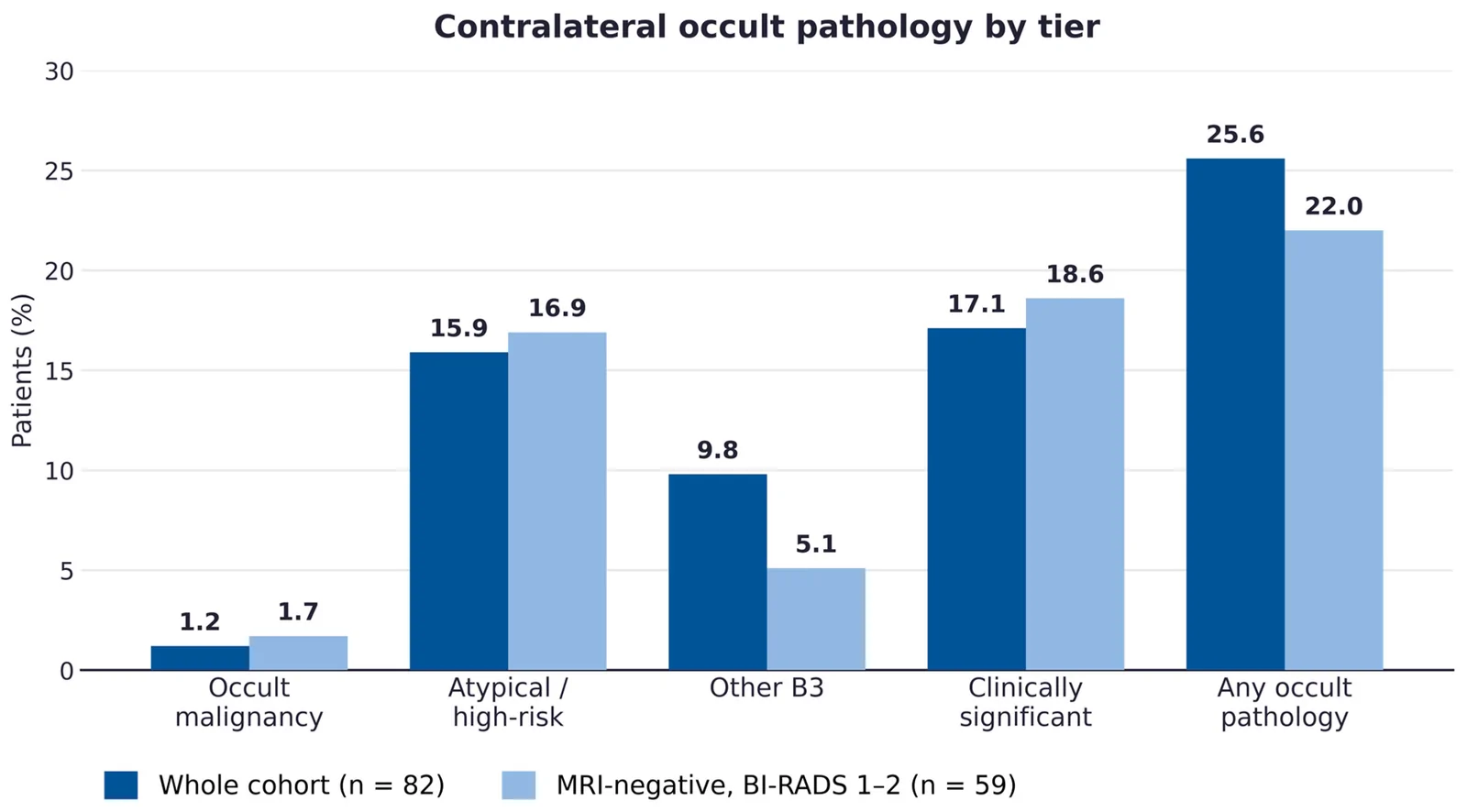
Contralateral occult pathology by tier in the whole cohort (*n* = 82) and the MRI-negative subgroup (BI-RADS 1–2, *n* = 59). The bars show the proportion of patients with each finding. The prevalence of atypical/high-risk lesions is similar in the whole cohort and the MRI-negative subgroup, indicating that these lesions are largely occult to MRI.

Occult malignancy (Tier 1) was identified in 1/82 patients (1.2%; 95% CI 0.0–6.6); this was a single focus of DCIS, and no occult invasive carcinoma was found (0/82; 95% CI 0.0–4.4). Atypical/high-risk lesions (Tier 2) were present in 13/82 patients (15.9%; 95% CI 8.7–25.6) and other B3 lesions (Tier 3) in 8/82 patients (9.8%). The primary outcome, clinically significant occult pathology (Tier 1 + Tier 2), was detected in 14/82 patients (17.1%; 95% CI 9.7–27.0), and any occult pathology (Tiers 1–3) was detected in 21/82 patients (25.6%). Benign incidental findings were common (44/82, 53.7%) but were not counted as occult pathology. Tier-specific rates are summarized in Table 2 and Figure 2.

### 3.3. Occult Pathology in the MRI-Negative Breast

Among the 59 patients with a negative contralateral MRI (BI-RADS 1–2), clinically significant occult pathology was found in 11 patients (18.6%; 95% CI 9.7–30.9), a rate descriptively similar to that observed in the whole cohort. Notably, the prevalence of atypical/high-risk lesions was similar in MRI-negative breasts (10/59, 16.9%) and in the whole cohort, consistent with the known tendency of such lesions to be non-enhancing and thus occult on MRI [15]. The results were consistent when an MRI-negative breast was instead defined as BI-RADS 1–3 (Table 2). The single occult malignancy also occurred in an MRI-negative breast: a 26-year-old BRCA1 carrier with a contralateral DCIS reported as BI-RADS 1. Patients with occult pathology in a MRI-negative breast are listed in Table 3.

### 3.4. Diagnostic Performance of Contralateral MRI

With BI-RADS ≥ 3 defined as a positive test, contralateral MRI showed low sensitivity for occult malignancy (0/1) because the only malignancy arose in an MRI-negative breast; the negative predictive value was 98.3% (95% CI 90.9–100.0), reflecting the rarity of malignancy rather than test performance. For clinically significant occult pathology, sensitivity was 21.4% and specificity 70.6%, with a positive predictive value of 13.0% and a negative predictive value of 81.4%. The diagnostic performance metrics are presented in Table 4. Because only one occult malignancy occurred, the sensitivity estimate for malignancy (0/1) is mathematically correct but clinically uninformative; all diagnostic performance estimates in this cohort, and those relating to malignancy in particular, should therefore be interpreted with caution.

### 3.5. Factors Associated with Occult Pathology

In the univariate exact analysis, the only biologically coherent factor nominally associated with clinically significant occult pathology was an index tumour DCIS component (whole cohort: OR 5.55, *p* = 0.020; MRI-negative subgroup: OR 9.69, *p* = 0.017). However, this association did not remain significant after the Benjamini–Hochberg correction (FDR-adjusted *p* = 0.19) and had wide confidence intervals. All clinically significant occult lesions occurred in premenopausal women (0/17 postmenopausal; *p* = 0.035, uncorrected), but this reflected a zero-cell distribution and did not survive correction. BRCA status, family history, age, breast density, and index tumour histology were not associated with occult pathology. No factor remained significant after correction for multiple comparisons; the full results are shown in Table 5. Several estimates in Table 5 are zero or infinite because one or more cells were empty; these odds ratios are unstable, reflect sparse data rather than strong associations, and should not be interpreted quantitatively.

## 4. Discussion

Two findings stand out in this cohort. Occult malignancy was rare, seen in only one of 82 women (1.2%). Atypical and high-risk lesions were not: they turned up in about one patient in six and just as often when the preoperative MRI had been read as negative. The useful point here is therefore less about occult cancer, which earlier work has already shown to be uncommon, than about these precursor lesions, since a clean contralateral MRI did little to lower the chance of finding them in the specimen.

Our single occult malignancy was an in situ lesion; no occult invasive carcinoma was identified (0/82; 95% CI 0.0–4.4). This is consistent with, and if anything, lower than the pooled incidence of 1.55% reported in the recent systematic review of Sturz-Ellis et al., in which occult malignancy was defined as invasive disease only [14]; including DCIS, our overall occult malignancy rate was 1.2%. A low rate was not a given in this cohort: the patients were young (median age of 42 years) and predominantly premenopausal, and a BRCA1/2 pathogenic variant was present in roughly one third of those tested (19/60), a risk profile well-described in young, BRCA-associated breast cancer [21,22] and one in which more occult disease might have been expected [13]. That the yield was nonetheless this low supports the broader argument that, once modern preoperative imaging has been performed, CPM adds little in terms of cancer detection. Notably, the one malignancy we did find arose in a breast reported as BI-RADS 1 so that a negative contralateral MRI, although reassuring, did not exclude occult carcinoma in this series.

These prevalence estimates should be read in the light of who was operated on. Every patient in this cohort had the risk profile described above and had also undergone preoperative MRI at a dedicated breast clinic. Rates from such a cohort cannot be applied directly to average-risk women with unilateral breast cancer, in whom the reasons for choosing CPM and the underlying probability of occult disease are both different. The same limitation applies in the opposite direction. In cohorts made up only of carriers of pathogenic variants, occult malignancy has been reported in approximately 4% of prophylactic mastectomies, falling to 3% when the preoperative MRI was BI-RADS 1–2 [13], and in up to 11.3% of specimens despite preoperative MRI [7]. Our results are therefore best understood as describing an intermediate group: an MRI-screened population that is increasingly seen in current practice.

The high negative predictive value of preoperative MRI for contralateral cancer is well established [11,12], and our data are consistent with it. They also show where it stops. Invasive or in situ cancer was uncommon, yet MRI-negative breasts still harboured atypical and high-risk lesions almost as often as the cohort as a whole. MRI is designed to find cancer rather than precursor lesions, and that is essentially what we observed.

The frequency of atypical and high-risk lesions we observed is broadly in keeping with earlier prophylactic mastectomy series, although lesion definitions, denominators and sampling differ between studies. Among 407 patients undergoing CPM between 1997 and 2005, King et al. reported occult contralateral carcinoma in 6% and a high-risk lesion in 28% [23]; Mattos et al. found malignancy in 7.0% and moderate- to high-risk lesions in 34.9% of CPM specimens [24]; and in bilateral risk-reducing mastectomy specimens, flat epithelial atypia, atypical ductal hyperplasia and lobular neoplasia were each described in 14–17% of patients [25]. Our composite atypical/high-risk rate of 15.9% therefore sits at the lower end of what these series report. The two higher figures come from series that used broader composite definitions of a high-risk lesion, whereas the study reporting each atypia category separately found rates closest to our own. Our malignancy rate, by contrast, is lower by several fold, which is what would be expected once every patient has undergone preoperative MRI: the modality removes much of the occult carcinoma from the specimen but leaves the precursor lesions behind. Thompson et al. observed the same directional effect, with preoperative MRI associated with a lower likelihood of occult malignancy in their contralateral prophylactic mastectomy cohort, an association not seen in their bilateral prophylactic mastectomy cohort [26].

The underlying biology offers a plausible explanation. Many B3/precursor lesions have non-specific or absent imaging correlates on dynamic contrast-enhanced MRI; when present, enhancement is typically non-mass and indistinguishable from benign parenchyma. Consequently, the value of MRI in this setting lies in its high negative predictive value for malignancy rather than in reliable depiction of these precursor lesions [15,18]. Flat epithelial atypia in particular tends to be non-enhancing [18]. Such lesions act as markers of future risk rather than as hidden cancers. Atypical hyperplasia and lobular neoplasia are well-recognized risk factors that raise a woman’s long-term chance of breast cancer [19,20], which is why finding them, even incidentally, can matter for how she is counselled and followed, even though on their own they seldom justify CPM. To our knowledge, the persistence of these lesions specifically in the MRI-negative breast has not been shown this directly before.

The value of MRI in this setting may therefore lie as much in what it cannot show as in what it can. These findings should not be interpreted as a limitation of MRI itself, but rather as a reflection of the biological and imaging characteristics of precursor lesions [15].

What, then, should follow when an atypical or high-risk lesion is reported in a CPM specimen? Within the CPM setting itself, usually little: the tissue at risk has been removed, so the lesion largely loses its predictive meaning for that breast, no re-excision is indicated, and most of these women are already receiving adjuvant endocrine therapy for an ER-positive index tumour (81.7% of our cohort, and all 14 women with clinically significant occult pathology), which is also the established risk-reducing intervention for atypical hyperplasia and lobular neoplasia [19,20]. Lobular neoplasia may nonetheless prompt review of the ipsilateral breast and chest wall. In a woman who retains her contralateral breast, the same diagnosis would carry a well-described long-term excess risk: a relative risk of approximately four for atypical hyperplasia, corresponding to a cumulative incidence of about 30% at 25 years [19], and a reported 7- to 10-fold increase for lobular carcinoma in situ, with an observed incidence of about 2% per year [20]. Either finding would justify a discussion of risk-reducing endocrine therapy, which substantially reduced subsequent cancer incidence in both settings [19,20], and, in selected cases, supplemental MRI surveillance [18,19], although a benefit from MRI screening has not been demonstrated in lobular carcinoma in situ specifically [20]. The more important implication is prospective and applies before surgery: because these lesions are common and largely invisible on MRI, a normal contralateral MRI should not be presented to a woman as evidence that her other breast is free of risk-relevant pathology; equally, the prospect of finding such a lesion is not in itself a reason to remove the breast, since the finding seldom alters management once the breast has been removed.

For counselling, this argues against using the prospect of occult malignancy as a reason to offer CPM. The decision still belongs where guidelines place it, resting on genuine clinical indications, hereditary risk, and the woman’s own preferences, and it should be reached together rather than dictated [1,2,3]. Decision aids can help structure that conversation [27]. This is consistent with the wider evidence on the operation itself: in a matched analysis of more than 100,000 women in the Surveillance, Epidemiology, and End Results registry, bilateral mastectomy markedly reduced the incidence of contralateral breast cancer but was not associated with lower breast cancer mortality over 20 years [28], and rates of the operation have continued to rise largely among women at no particular risk of contralateral disease [29]. Our results add to the recent Society of Surgical Oncology Working Group statement [3] by showing that even among women who choose CPM after MRI, the pathological yield of occult cancer is very small.

None of the variables we examined, whether clinical, imaging, or genetic, predicted clinically significant occult pathology once we corrected for multiple testing. An index tumour with a DCIS component came closest, but the signal did not survive correction and is best regarded as hypothesis-generating; predictors of occult pathology in this setting warrant dedicated study [26]. In practice, routine preoperative features are not enough to reliably identify the women at negligible risk. Reliable predictors are unlikely to emerge from single-centre series of this size; multicentre cohorts or registry-based analyses, with prospectively standardized pathological sampling, will be required to test the associations suggested here.

Clinically, these findings carry a cautious corollary. In a woman who declines CPM, a normal contralateral MRI should not be taken as proof that nothing is present, particularly if she is young or carries a mutation. Continued imaging and individual risk assessment remain central to her care.

The study has genuine strengths. All operations were performed by a single surgeon at a dedicated breast clinic, and all specimens were reviewed by pathologists experienced in breast pathology, providing consistency in surgical technique and pathological assessment. Every woman had a preoperative contralateral MRI, which let us look at the MRI-negative breast directly. We used a predefined three-tier scheme and exact statistics with correction for multiple testing, which guards against reading too much into chance findings.

### Limitations

The study also has clear limits. Its retrospective, single-centre design carries the biases inherent to that approach: patients were not selected at random, the data were generated for clinical rather than research purposes, and both the decision to perform CPM and the decision to obtain MRI were taken in routine practice, so selection and indication bias cannot be excluded. Unmeasured confounders, such as the degree of cancer-related anxiety or the intensity of previous imaging, were not available. Because all operations were performed by a single surgeon at a dedicated breast clinic, the internal consistency this affords is offset by limited external validity: referral to such a practice is likely to select for younger, genetically tested and highly motivated women, and both the case mix and the threshold for offering CPM may differ elsewhere. The study database was assembled from records of bilateral mastectomy, so the proportion of the clinic’s total mastectomy volume represented by this cohort could not be determined and the degree of selection within the practice cannot be quantified. Our estimates should therefore be read as descriptive of this setting and require confirmation in multicentre cohorts.

With only one occult malignancy, predictors of malignancy could not be modelled, and MRI’s performance for cancer rests on a single event; all predictor analyses were exploratory, with wide confidence intervals and nothing surviving correction. Specimens were reported by different pathologists in routine practice, without central review or formal inter-observer assessment; although sampling was systematic, it was not fixed to a set number of blocks, and more extensive sampling would probably have increased the yield of atypia. All examinations performed at the single dedicated breast imaging centre were acquired at 1.5 T, and the equipment used for the six studies obtained at external centres is not known. Since 3.0 T offers a higher signal-to-noise ratio and has been reported to correlate better than 1.5 T with the pathological extent of disease [30,31], some of the lesions we classified as occult might have been detected at higher field strength, and our findings should be read as applying to contralateral MRI performed at 1.5 T. BRCA testing was performed in 60 of the 82 patients and was not applied systematically, so both the carrier prevalence and the analysis of BRCA status as a predictor rest on a selected subgroup, and our composite primary outcome spans lesions of differing clinical weight, from ductal carcinoma in situ to flat epithelial atypia, for which Table 2 gives the tier-specific rates. Finally, we had no comparison group of women who declined CPM and no long-term follow-up or survival data, so the clinical consequences of the lesions we identified could not be measured directly.

## 5. Conclusions

Among women undergoing CPM after a preoperative contralateral MRI, occult malignancy was rare (1.2%). A negative MRI went with a very low rate of occult cancer but did not rule it out completely; the one cancer we found lay in a breast read as BI-RADS 1. Clinically significant occult pathology, mostly atypical and high-risk lesions, was present in about 17% of women and was largely invisible to MRI, occurring at much the same rate whether or not the scan was negative. No routine clinical or tumour feature reliably flagged these findings in advance. The decision to perform CPM is therefore better grounded in individual risk and shared decision-making than in any expectation of what pathology the specimen might reveal.

## Figures and Tables

**Table 1:** Cohort characteristics and association with clinically significant occult pathology (Tier 1 + 2).

Variable	Whole Cohort (*n* = 82)	Occult+ (*n* = 14)	Occult− (*n* = 68)	*p*
Age, median (IQR)	42 (38–48)	45	42	0.781
BMI, median	24	23	24	0.635
Postmenopausal	17/82 (20.7%)	0/14 (0%)	17/68 (25%)	0.035
BRCA1/2-positive *	19/60 (31.7%)	1/11	18/49	0.148
First-degree family history	19/82 (23.2%)	1/14 (7%)	18/68 (26%)	0.171
Second-degree family history	27/82 (32.9%)	6/14 (43%)	21/68 (31%)	0.533
Index tumour DCIS component	47/82 (57.3%)	12/14 (86%)	35/68 (51%)	0.020
Index lobular (ILC/Mixed)	12/82 (14.6%)	3/14 (21%)	9/68 (13%)	0.422
Multifocal/multicentric	31/82 (37.8%)	8/14 (57%)	23/68 (34%)	0.133
ER-positive	67/82 (81.7%)	14/14 (100%)	53/68 (78%)	0.062
Dense breast (C/D)	65/82 (79.3%)	11/14 (79%)	54/68 (79%)	1.000

* Of 60 patients tested for BRCA. BMI available for 80 patients. Continuous variables, Mann–Whitney U test; categorical variables, Fisher’s exact test. Exploratory analysis.

**Table 2:** Contralateral occult pathology by three-tier classification (95% CI; *n*).

Outcome Tier	Whole Cohort (*n* = 82)	MRI-Negative Subgroup (BI-RADS 1–2) (*n* = 59)
Tier 1—Occult malignancy (invasive + DCIS)	1.2% (0.0–6.6); *n* = 1	1.7% (0.0–9.1); *n* = 1
Tier 2—Atypical/high-risk (ADH/ALH/LCIS/FEA/atypical papilloma)	15.9% (8.7–25.6); *n* = 13	16.9% (8.4–29.0); *n* = 10
Tier 3—Other B3 (radial scar/CSL/papilloma without atypia)	9.8% (4.3–18.3); *n* = 8	5.1% (1.1–14.1); *n* = 3
**Clinically significant occult pathology (Tier 1 + 2)**	**17.1% (9.7–27.0); *n* = 14**	**18.6% (9.7–30.9); *n* = 11**
Any occult pathology (Tier 1 + 2 + 3)	25.6% (16.6–36.4); *n* = 21	22.0% (12.3–34.7); *n* = 13
Benign incidental (FA/apocrine metaplasia/sclerosing adenosis)	53.7% (42.3–64.7); *n* = 44	55.9% (42.4–68.8); *n* = 33

Clopper–Pearson exact 95% CI. DCIS is reported under the malignancy tier. Benign incidental findings were not counted as occult pathology. Bold indicates the primary outcome.

**Table 3:** Patients with occult pathology in an MRI-negative (BI-RADS 1–2) breast (*n* = 13).

MRI BI-RADS	Index Histology	BRCA	Age	Tier	Contralateral Finding
1	IDC	BRCA1	26	T1	DCIS
1	ILC	-	40	T2	Radial scar, Atypical papilloma
1	IDC	-	49	T2	ALH
1	IDC	-	45	T2	ADH, FEA
1	IDC	-	47	T2	FEA, Atypical papilloma
1	IDC	-	47	T2	ADH
1	IDC	-	44	T2	ALH, LCIS
2	IDC	-	48	T2	ADH
2	IDC	-	39	T2	FEA
2	IDC	-	42	T2	ADH, ALH
2	IDC	-	40	T2	ADH
2	IDC	-	50	T3	Papilloma
2	IDC	BRCA2	42	T3	Radial scar, CSL, Papilloma

Names removed for confidentiality. Tier: T1 = malignancy, T2 = atypical/high-risk, T3 = other B3.

**Table 4:** Diagnostic performance of contralateral MRI (BI-RADS ≥ 3 as positive).

Metric	Occult Malignancy (T1)	Clinically Significant (T1 + 2)	Any Occult (T1 + 2 + 3)
Sensitivity	0.0% (0.0–97.5)	21.4% (4.7–50.8)	38.1% (18.1–61.6)
Specificity	71.6% (60.5–81.1)	70.6% (58.3–81.0)	75.4% (62.7–85.5)
Positive predictive value	0.0% (0.0–14.8)	13.0% (2.8–33.6)	34.8% (16.4–57.3)
Negative predictive value	98.3% (90.9–100.0)	81.4% (69.1–90.3)	78.0% (65.3–87.7)

Values are % (Clopper–Pearson 95% CI). The single occult malignancy occurred in an MRI-negative breast; MRI sensitivity (0/1) should not be interpreted on a single event.

**Table 5:** Predictors of clinically significant occult pathology (exact method, FDR-corrected).

Whole Cohort (*n* = 82, Events = 14)					
Variable	Occult+ Rate	Exact OR	95% CI	*p*	*p* (FDR)
Index tumour DCIS component	26% vs. 6%	5.55	1.11–54.83	0.020 *	0.192
Postmenopausal	0% vs. 22%	0.00	0.00–1.04	0.035 *	0.192
ER-positive	21% vs. 0%	∞	0.81–∞	0.062	0.228
Multifocal/multicentric	26% vs. 12%	2.58	0.69–10.19	0.133	0.313
BRCA1/2-positive	5% vs. 21%	0.22	0.00–1.65	0.171	0.313
First-degree family history	5% vs. 21%	0.22	0.00–1.65	0.171	0.313
HRT history	24% vs. 15%	1.79	0.41–7.05	0.335	0.527
Lobular (ILC/Mixed)	25% vs. 16%	1.77	0.27–8.77	0.422	0.580
Second-degree family history	22% vs. 15%	1.67	0.42–6.30	0.533	0.652
Genetic mutation	15% vs. 20%	0.71	0.18–2.62	0.770	0.847
Dense breast (C/D)	17% vs. 18%	0.95	0.21–6.02	1.000	1.000
**MRI-Negative Subgroup, BI-RADS 1–2 (*n* = 59, Events = 11)**					
**Variable**	**Occult+ Rate**	**Exact OR**	**95% CI**	***p***	***p* (FDR)**
Index tumour DCIS component	29% vs. 4%	9.69	1.21–451.65	0.017 *	0.190
ER-positive	23% vs. 0%	∞	0.70–∞	0.098	0.475
HRT history	31% vs. 14%	2.75	0.55–13.34	0.149	0.475
Postmenopausal	0% vs. 22%	0.00	0.00–1.86	0.183	0.475
BRCA1/2-positive	6% vs. 23%	0.22	0.00–1.85	0.259	0.475
First-degree family history	7% vs. 23%	0.25	0.01–2.05	0.259	0.475
Genetic mutation	13% vs. 24%	0.49	0.09–2.23	0.333	0.524
Multifocal/multicentric	23% vs. 15%	1.67	0.37–7.95	0.511	0.703
Second-degree family history	21% vs. 18%	1.25	0.23–5.87	0.734	0.897
Dense breast (C/D)	19% vs. 19%	0.99	0.20–6.68	1.000	1.000
Lobular (ILC/Mixed)	14% vs. 19%	0.70	0.01–6.93	1.000	1.000

Exact conditional OR; “∞” denotes a zero-cell (separation) and is not interpretable. * *p* < 0.05 before correction for multiple comparisons. No variable remained significant after FDR correction.

## Data Availability

The data presented in this study are available on request from the corresponding author. The data are not publicly available due to privacy and ethical restrictions.

## Supplementary Materials

The following supporting information can be downloaded at: https://www.mdpi.com/article/10.3390/cancers18162710/s1, Table S1: STROBE Statement—Checklist of items that should be included in reports of cohort studies.
