# Supplementary material for: Occult Pathology in the Contralateral Prophylactic Mastectomy Specimen Despite a Negative Contralateral MRI: A Single-Center Cohort Study
Source: Cancers (Basel). 2026 Aug 21;18(16):2710. doi: 10.3390/cancers18162710 (PMC13511345; doi:10.3390/cancers18162710)
Supplement: Supplementary file 1 [file cancers-18-02710-s001.zip › cancers-4478835-supplementary.pdf]

Occult Pathology in the Contralateral Prophylactic Mastectomy Specimen Despite a Negative Contralateral MRI: A Single-Center Cohort Study

Table S1. STROBE Statement — Checklist of items that should be included in reports of cohort studies.

Item	Recommendation	Reported in / Section
1	Title and abstract. (a) Indicate the study's design in the title or abstract. (b) Provide a balanced summary of what was done and found.	Title ("…Cohort Study"); Abstract (structured: Background/Methods/Results/Conclusions)
**INTRODUCTION**		
2	Background/rationale. Explain the scientific background and rationale.	Introduction, paragraphs 1–3
3	Objectives. State specific objectives, including any prespecified hypotheses.	Introduction, final paragraph (aims i–iii)
**METHODS**		
4	Study design. Present key elements of study design early in the paper.	Section 2.1 (single-centre, retrospective cohort)
5	Setting. Describe setting, locations, and relevant dates of recruitment, exposure, follow-up, and data collection.	Section 2.1 (breast surgery clinic; January 2022–December 2025)
6	Participants. (a) Give eligibility criteria, and the sources and methods of selection of participants.	Section 2.2; Figure 1 (patient selection flow)
7	Variables. Clearly define all outcomes, exposures, predictors, potential confounders, and effect modifiers.	Sections 2.3 (three-tier classification; primary/secondary outcomes) and 2.4 (MRI/BI-RADS definitions)
8	Data sources/measurement. For each variable, give sources of data and details of methods of assessment.	Sections 2.3 (pathology; WHO classification) and 2.4 (MRI protocol; ACR BI-RADS)

Item	Recommendation	Reported in / Section
9	Bias. Describe any efforts to address potential sources of bias.	Sections 2.4 (predefined MRI-negative definition; sensitivity analysis) and 2.5 (exact methods; FDR correction; exploratory framing)
10	Study size. Explain how the study size was arrived at.	Section 2.2; Figure 1 (all eligible consecutive patients in the period; n = 82)
11	Quantitative variables. Explain how quantitative variables were handled in the analyses.	Section 2.5 (median/IQR; categorical counts/percentages)
12	Statistical methods. (a) Describe all statistical methods, including those used to control for confounding. (b) Subgroups/interactions. (c) Missing data. (d) Loss to follow-up. (e) Sensitivity analyses.	Section 2.5 (Clopper-Pearson exact CI; Fisher; Mann-Whitney U; exact conditional OR; Benjamini-Hochberg FDR; predefined MRI-negative subgroup; BI-RADS 1–3 sensitivity analysis; no multivariable modelling given low event counts)
**RESULTS**		
13	Participants. (a) Numbers at each stage. (b) Reasons for non-participation. (c) Consider a flow diagram.	Section 3.1; Section 2.2; Figure 1
14	Descriptive data. (a) Characteristics of participants and information on exposures/confounders. (b) Missing data. (c) Follow-up time.	Section 3.1; Table 1 (BMI available for 80 patients noted)
15	Outcome data. Report numbers of outcome events or summary measures.	Sections 3.2–3.3; Tables 2 and 3; Figure 2

Item	Recommendation	Reported in / Section
16	Main results. (a) Unadjusted estimates and, where applicable, confounder-adjusted estimates and their precision (e.g., 95% CI). (b) Category boundaries. (c) Absolute risk where relevant.	Sections 3.2–3.5; Tables 2–5 (proportions with exact 95% CI; exact OR with 95% CI; FDR-adjusted p)
17	Other analyses. Report other analyses done (subgroups, interactions, sensitivity analyses).	Section 3.3 (MRI-negative subgroup; BI-RADS 1–3 sensitivity analysis); Section 3.4 (diagnostic performance)
**DISCUSSION**		
18	Key results. Summarise key results with reference to study objectives.	Discussion, paragraph 1
19	Limitations. Discuss limitations, taking into account sources of potential bias or imprecision.	Section 4.1 (Limitations)
20	Interpretation. Give a cautious overall interpretation considering objectives, limitations, multiplicity of analyses, and other relevant evidence.	Discussion, paragraphs 2–7; Conclusions
21	Generalisability. Discuss the generalisability (external validity) of the study results.	Section 4.1 (single-centre; cohort enriched for young, premenopausal, mutation carriers; all MRI-screened)
**OTHER INFORMATION**		
22	Funding. Give the source of funding and the role of funders.	This research received no external funding.

Page/line numbers refer to the submitted manuscript. An Explanation and Elaboration article discusses each checklist item and gives methodological background and published examples of transparent reporting. The STROBE checklist is best used in conjunction with this article (freely available at www.strobe-statement.org (accessed on 17 August 2026)).
